# Experimental Design-Driven Optimization of Choline-Based Natural Deep Eutectic Solvents for the Extraction of Bioactive Compounds from *Bellevalia dubia*

**DOI:** 10.3390/molecules31183267

**Published:** 2026-09-15

**Authors:** Anastasia Charalampidopoulou, Marie Zlechovcová, Charalampos Proestos, Chrysavgi Gardeli, Aristeidis S. Tsagkaris

**Affiliations:** 1 Laboratory of Food Chemistry and Analysis, Department of Food Science and Human Nutrition, Agricultural University of Athens, Iera Odos 75, 11855 Athens, Greece; 2 Department of Food Analysis and Nutrition, Faculty of Food and Biochemical Technology, University of Chemistry and Technology Prague, Technická 5, 166 28 Prague, Czech Republic; 3 Food Chemistry Laboratory, Department of Chemistry, National and Kapodistrian University of Athens, 15772 Athens, Greece

**Keywords:** enzyme-inhibition assays, experimental design, high-resolution mass spectrometry, deep eutectic solvents, bioactivity

## Abstract

The development of sustainable and efficient sample-preparation strategies remains a key challenge in analytical workflows for plant-derived bioactive compounds. In this study, choline chloride (ChCl)-based natural deep eutectic solvents (NADES) were systematically investigated as green extraction media and combined with chemical and enzyme assays to identify the bioactive effect of phenolic compounds from *Bellevalia dubia*, an understudied Mediterranean plant. A design of experiments approach was applied to evaluate the influence of hydrogen bond donors (lactic, malic, and citric acid) and their molar ratios on extraction performance. Among the tested systems, the ChCl–malic acid (MA) mixture at a molar ratio of 1:2.5 (precisely 1:2.47) was identified as the best overall solution according to the desirability approach, which integrated both phytochemical and enzyme-inhibitory responses. This system also showed strong enzyme-inhibitory effects compared to conventional extractants (water and 80% methanol). To identify the extract composition, a suspect screening workflow was applied based on ultra-high-performance liquid chromatography hybrid quadrupole Orbitrap mass spectrometry (UHPLC–q-Orbitrap-MS). Twenty-one compounds were detected and annotated, mainly belonging to the flavonoid class. Principal component analysis (PCA) showed that ChCl-MA extracts were discriminated, particularly at molar ratio 1:2.5, from the rest, indicating a distinct chemical and bioactivity profile. PCA further suggested a possible association of the ChCl-MA 1:2.5 system with vitexin and AChE inhibition, which requires further investigation. Overall, the proposed NADES-based approach offers a tunable, promising, and potentially more sustainable alternative for the extraction of bioactive compounds from plant-based sources.

## 1. Introduction

*Bellevalia dubia* belongs to the *Asparagaceae* family and it grows primarily in the Mediterranean area. The flowers of *B. dubia* are a vivid, deep shade of blue and are arranged in racemose inflorescences. The scientific literature describing the phytochemical profile and biological properties of *B. dubia* remains limited. Consequently, the findings of the present study serve to address these critical knowledge gaps and establish an empirical baseline for the species. Its secondary metabolite composition and bioactive potential are currently inferred via chemotaxonomic extrapolation from well-characterized congeners such as *Muscari*, *Hyacinthoides*, *Scilla*, and *Brimeura* [1]. Specifically, El-Elimat et al. [2] identified four new homoisoflavonoids, particularly dihydropunctatin derivatives, which exhibited potent activity against different cancer cell lines. In addition, Yolbaş [3] reported that *B. pseudolongipes* demonstrated high levels of antioxidant activity, with caffeic acid, vitexin, schaftoside, orientin, and narirutin being the predominant compounds. Ouelbani et al. [4] showed that the ethyl acetate crude extract from bulbs and roots exhibited moderate antibacterial activity, along with noticeable acetylcholinesterase (AChE) inhibition and antioxidant activity. *M. comosum* is the most closely related genus to *B. dubia*, and several studies have investigated its phytochemical composition as well as its biological properties. Afshar et al. [5] reported that the chloroform extract showed significant antioxidant activity, likely related to the presence of flavonoids and coumarins. Additionally, Casacchia et al. [6] reported that *M. comosum* extracts inhibited pancreatic lipase (PNLIP) and *α*-amylase activities in vitro, while administration of the extracts to high-fat-diet-fed rats improved several metabolic and anthropometric parameters, supporting their potential role in preventing obesity-related metabolic disorders.

In recent years, deep eutectic solvents (DES) have emerged as promising green alternatives due to their low toxicity, reduced environmental impact, and tunable physicochemical properties that enhance extraction selectivity [7]. DES are formed by combining a hydrogen bond acceptor (HBA) and a hydrogen bond donor (HBD), resulting in mixtures with significantly lower melting points than their individual components [8]. A subclass, natural deep eutectic solvents (NADES), has shown improved extraction efficiency and biocompatibility, making them suitable for applications in food, pharmaceutical, and cosmetic fields. NADES are regarded as “natural” because their constituents are primarily naturally occurring compounds found in plants, including sugars, organic acids and bases, and amino acids [9]. Among them, choline chloride-based NADES are the most widely studied, with their properties easily tuned by selecting a different HBD [10].

The polarity of NADES depends on the nature of their components and, therefore, plays a decisive role in extraction efficiency [9]. According to the literature, organic acid-based NADES are particularly effective for the extraction of phenolic compounds [11,12]. NADES containing organic acids such as citric acid (CA), malic acid (MA), or lactic acid (LA) exhibit high polarity due to the presence of multiple carboxyl groups, which enhance hydrogen-bonding interactions with the target compounds [9]. In this context, Bosiljkov et al. [13] demonstrated that the ChCl-MA system achieved superior recovery of anthocyanins from wine lees compared to conventional solvents.

However, the high viscosity of DES/NADES can limit mass transfer and influence extraction selectivity. Hydrogen bonding, electrostatic interactions, and van der Waals forces play a key role in determining their viscosity. DES/NADES typically exhibit viscosities that are 100–1000 times higher than those of water and conventional organic solvents due to the strong hydrogen-bonding network between their components. This also restricts the movement of free species within the solvent system [9,14]. NADES based on polyols exhibit lower viscosity than those based on acids and sugars, likely due to their smaller molecular size, which leads to weaker intermolecular interactions and less extensive hydrogen-bonding networks [8,15].

Adding water weakens the hydrogen-bonding network, increases osmotic pressure, enhances mass transfer, and reduces viscosity. This effect is most beneficial at moderate water content levels, typically between 20% and 30% (*v*/*v*). However, at concentrations above 50% (*v*/*v*), hydrogen-bond interactions are significantly disrupted or lost. This causes the DES to lose its structural integrity and behave as a mixture of separate HBA and HBD components [16,17]. The combination of green solvents such as DES/NADES with energy-assisted extraction techniques represents a promising approach to maximize the recovery of phenolic and antioxidant compounds while reducing environmental impact [10].

In the present study, a design-based approach was applied to systematically evaluate different NADES composition and molar ratios. The experimental runs generated by the design software were evaluated through a desirability function, allowing the simultaneous consideration of multiple responses and the statistical identification of the most suitable extraction system based on the overall desirability value. Organic acids were selected as HBD to match the polarity of phenolic compounds, while 30% (*w*/*w*) water was added to reduce viscosity and enhance extraction efficiency. First, the extracts were evaluated for total phenolic and flavonoid content (TPC and TFC, respectively), antioxidant activity, and color stability. Enzyme assays were then conducted to assess inhibitions against AChE and PNLIP, which are associated with cognitive function and dietary lipid metabolism, respectively. Bioactive compounds were characterized using ultra-high-performance liquid chromatography hybrid quadrupole Orbitrap mass spectrometry (UHPLC-q-Orbitrap-MS) through a suspect screening. Finally, the optimal composition of the NADES was determined by maximizing the overall desirability function.

## 2. Results and Discussion

### 2.1. Chemical Assays

**Figure 1:**
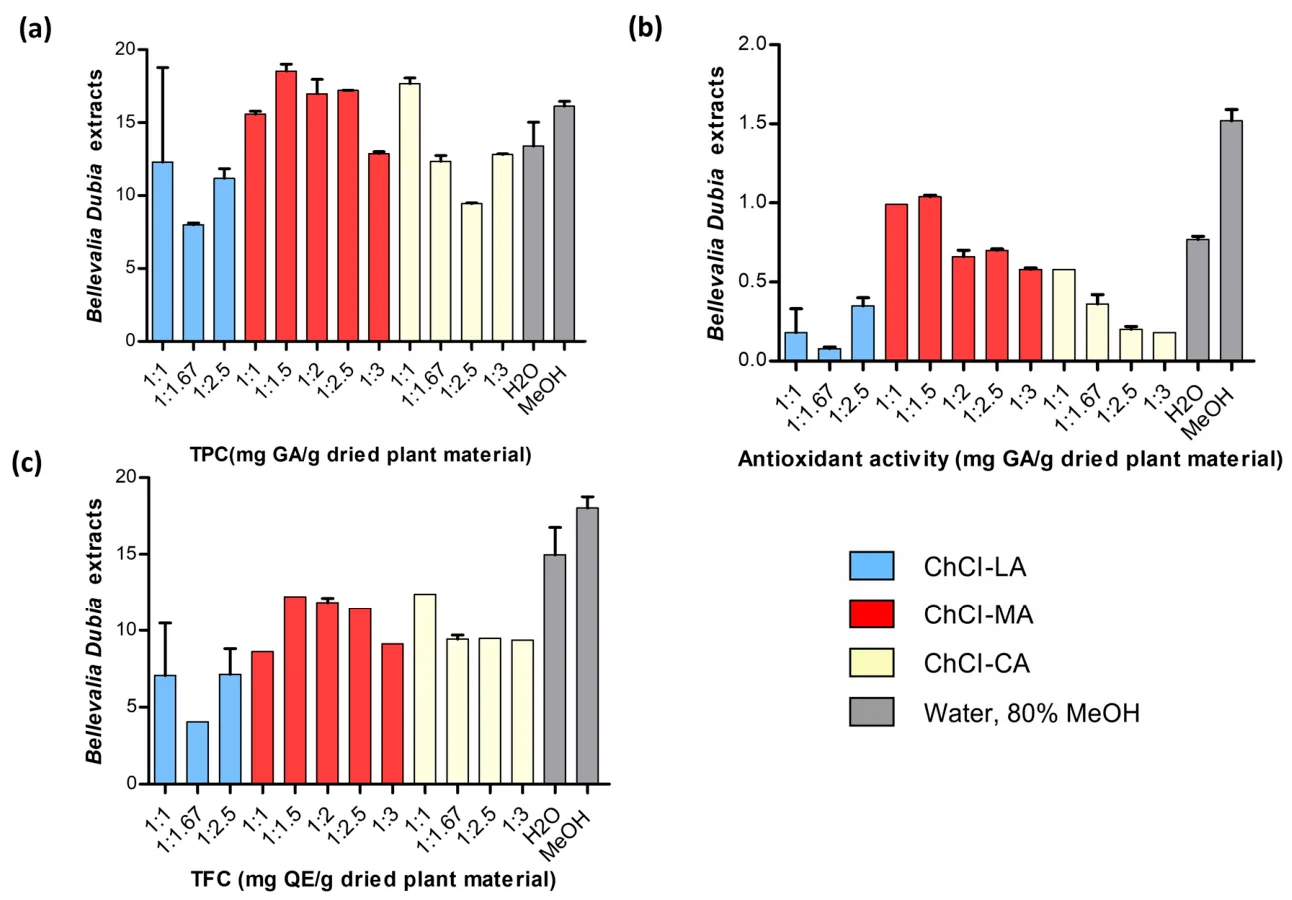
(**a**) TPC, (**b**) antioxidant activity, and (**c**) TFC of *B. dubia* flower extracts obtained using NADES and conventional solvents (80% MeOH:H_2_O and H_2_O). Concentrations are expressed in mg GAE/g or mg QE/g dry plant material, as appropriate. Values are expressed as mean ± SD (*n* = 2) for TPC and DPPH. TFC values represent single analytical determinations. More info is provided in the Supplementary Material, Table S1.

Clear differences were noticed for TPC, TFC, and antioxidant activity, depending on the solvent system used (Figure 1). Particularly, NADES based on ChCl-MA (1:1.5) (18.52 ± 0.48 mg GAE/g) and ChCl-CA (1:1) (17.68 ± 0.38 mg GAE/g) yielded the highest TPC. In terms of conventional solvents, 80% methanol extracted a greater number of total phenolics (16.13 ± 0.33 mg GAE/g) than water (13.39 ± 1.63 mg GAE/g) (Figure 1a). This difference was expected due to the intermediate polarity of methanol, which dissolves a broader range of phenolic compounds more effectively than water. The superior extraction efficiency observed for NADES systems can likely be explained by the synergistic effects of hydrogen-bond interactions between the NADES components and the hydroxyl groups of phenolic compounds [9].

However, the conventional solvents (water and 80% methanol) resulted in higher flavonoid yields than the NADES extracts, with 80% methanol showing the highest TFC (18.02 ± 0.73 mg QE/g), followed by water (14.97 ± 1.79 mg QE/g). As shown in Figure 1c, among the NADES systems, the ChCl-MA (1:1.5) and ChCl-CA (1:1) systems showed the best performance, yielding 12.23 mg QE/g and 12.39 mg QE/g, respectively, whereas the ChCl-LA system resulted in comparatively lower flavonoid recovery. Organic-based NADES containing di- or tri-carboxylic acids (e.g., MA and CA) form strong hydrogen-bond networks and exhibit high viscosity, which can limit solute interaction and mass transfer, possibly explaining the reduced extraction efficiency compared to conventional solvents [18].

The highest antioxidant activity was observed for the 80% methanol extract (1.52 ± 0.07 mg GAE/g), as shown in Figure 1b. This was followed by the ChCl-MA systems at ratios of 1:1.5 and 1:1, which yielded 1.04 and 0.99 mg GAE/g, respectively. Water showed a noticeably lower antioxidant activity, with a value of 0.77 ± 0.02 mg GAE/g. The superior performance of 80% methanol extract can likely be attributed to its greater efficiency in extracting flavonoids, compounds that are well known for their contribution to radical scavenging activity [19].

Previous studies confirm that solvent polarity and extraction efficiency strongly influence both phenolic yield and antioxidant performance in *M. comosum* (closest genus to *B. dubia* with available data). Traditional organic solvents such as methanol and ethanol have generally been regarded as optimal for recovering bioactive polyphenols, with some extracts showing remarkably low IC_50_ values (e.g., 0.0367 mg/mL in the methanolic extract of *M. comosum* reported by Larocca et al. [20], and 0.0409 mg/mL in the ethanolic extract described by Loizzo et al. [21]. However, despite their extraction efficiency, the use of volatile organic solvents raises concerns regarding environmental impact, compound stability, and selectivity toward hydrophilic antioxidants.

Previous studies have reported values of 6.28 ± 0.23 mg GAE/g for *M. inconstrictum* and 15.11 ± 0.78 mg GAE/g for methanolic extracts of *M. comosum* [5,22]. A moderate antioxidant activity has also been reported by Marrelli et al. in extracts of related species [23,24]. Also, significant levels of flavonoids were reported in both methanolic and ethanolic extracts of *M. comosum*. In addition, *B. mauritanica* extracts were also shown to have high phenolic and flavonoid contents and promising antioxidant activity [4].

### 2.2. Color Stability of NADES Extracts

The half-life and destruction time values for the color degradation of *B. dubia* extracts obtained with different NADES are presented in Table 1. Only the systems that exhibited satisfactory linearity (R^2^ > 0.90) were included. The results showed noticeable variability among the NADES tested. The extract prepared with ChCl-CA (1:1.67) displayed the highest color stability, with a half-life of 51 days and a destruction time of 169 days, suggesting a slower degradation rate of pigments under these conditions. In contrast, the ChCl-MA (1:1.5) system exhibited the lowest stability, with a half-life of 14 days and a destruction time of 47 days. These results indicate that the type of HBD and the molar ratio both strongly affect pigment stability.

NADES containing CA appear to better preserve pigment color, possibly due to stronger intermolecular interactions and reduced pigment oxidation. This enhanced stability may also be linked to the higher viscosity of CA-based NADES, which can limit pigment diffusion and slow degradation kinetics [9]. Barbieri et al. [25] used the Weibull model to estimate the half-life of NADES extracts. The half time of the NADES extracts ranged from 7 to 49 days while the half-life of the ethanol extract was significantly lower. According to Liu et al. [26], curcumin exhibited much higher stability in a citric acid-glucose NADES than in ethanol and methanol at 80 °C. This improvement was attributed to the formation of strong hydrogen bonds between the hydroxyl groups of curcuminoids and the NADES components, which reduced oxygen contact and prevented oxidative damage.

### 2.3. The In Vitro Enzyme-Inhibitory Effect of B. dubia Extracts

Among the NADES systems, ChCl-MA systems exhibited the most pronounced inhibitory effects among the tested solvents (Figure S1), likely due to its more moderate polarity and viscosity relative to the other two ChCl–organic acid combinations. In particular, the ChCl-MA (1:2.5) extract demonstrated strong inhibition against both PNLIP (86.73 ± 9.75%) and AChE (78.21 ± 12.96%), followed by ChCl-MA (1:3), which also displayed notable activity. Overall, all NADES extracts produced strong or medium inhibition levels for PNLIP. On the other hand, the conventional 80% MeOH and aqueous extracts showed markedly lower inhibition under the same experimental conditions. The final concentration of all extracts for all extractants was 0.1 mg/mL in the AChE assay and 0.025 mg/mL in the PNLIP assay. In any case, further investigation is needed to identify the inhibitory potential of the extracts through dose–response experiments, and this represents a limitation of the present study.

Previous studies have reported variable inhibition levels depending on the solvent system and plant part used. For instance, Larocca et al. [20] observed moderate AChE inhibition for *M. comosum* methanolic extracts with an IC_50_ value of 0.1076 mg/mL. Furthermore, a study on *B. mauritanica* evaluating its antioxidant, antibacterial, and anticholinesterase activities reported that the EtOAc extract inhibited AChE with an IC_50_ of 7.09 ± 0.04 μg/mL, which is close to that of the standard galantamine (IC_50_: 6.27 ± 1.15 μg/mL; a known AChE inhibitor), indicating the plant’s strong neuroprotective potential [4].

Regarding PNLIP, *M. comosum* extracts have repeatedly demonstrated strong inhibitory potential. Marrelli et al. [24] reported that wild bulb extracts exhibited pronounced activity (IC_50_ = 0.166 ± 0.004 mg/mL), while leaf extracts showed a weaker response (IC_50_ = 3.819 ± 0.119 mg/mL) [23]. Casacchia et al. [27] observed that raw bulb extracts inhibited PNLIP by 58.82% at 0.25 mg/mL, whereas heat-treated samples (steamed) showed reduced activity. Notably, in another study, ethanolic extracts of *M. comosum* exhibited an IC_50_ of 0.0705 mg/mL against PNLIP, comparable to that of orlistat, confirming their strong anti-lipase potential [6].

The effectiveness of NADES media may be related to their polarity and hydrogen-bonding capacity, which could enhance the solubilization and stabilization of the bioactive compounds potentially involved in enzyme inhibition [9]. In any case, parameters including extraction temperature and duration, solvent-to-material ratio, and the application of assisted techniques can significantly influence both the yield and stability of phenolic compounds [28]. Consequently, these methodological differences should be evaluated alongside solvent effects when interpreting and comparing study outcomes.

### 2.4. Comparative Analysis of Different Solvent Systems Across Tested Assays

**Figure 2:**
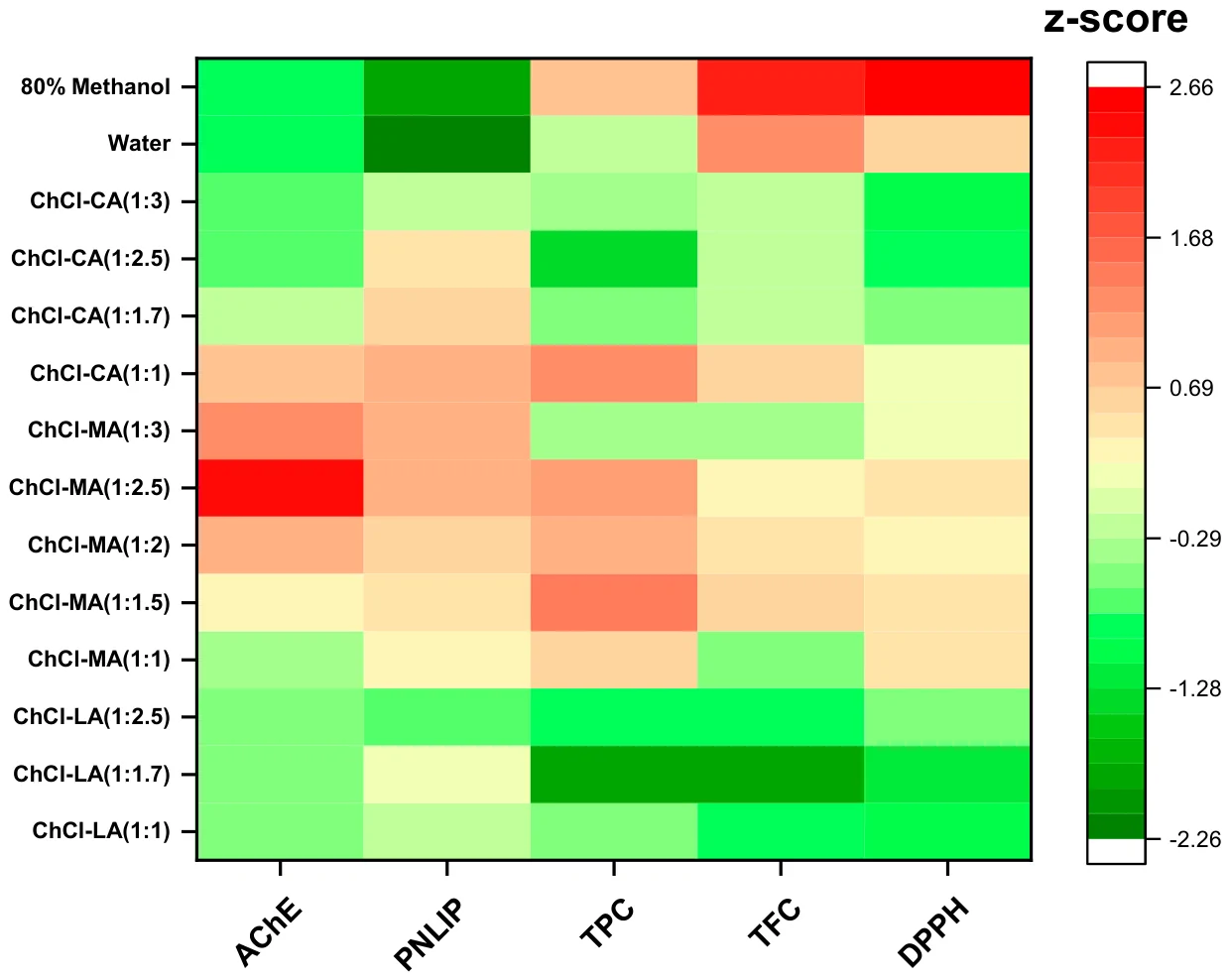
Heatmap showing the inhibition of AChE and PNLIP and the corresponding TPC, TFC, and DPPH values of *B. dubia* extracts obtained with different NADES and conventional solvents.

The heatmap, based on z-score normalization, provided an integrated overview of the performance of the extracts obtained by different solvent systems across chemical composition and bioactivity assays (Figure 2). A general trend was observed; extracts with higher phenolic and flavonoid content also exhibited enhanced antioxidant activity and, in some cases, increased enzyme inhibition. This pattern suggests a possible contribution of phenolic compounds to the observed bioactivities, in agreement with previous studies. Particularly, methanol extracts exhibited high relative values for TFC and DPPH, indicating their strong ability to extract flavonoids and other phenolic compounds associated with antioxidant activity. However, they showed lower PNLIP inhibitory activity. Among the NADES systems, ChCl-MA extracts demonstrated the most consistent performance, particularly at molar ratios 1:1.5, 1:2, and 1:2.5, where positive z-scores were observed across all evaluated assays. In contrast, ChCl-CA systems showed intermediate behavior, with moderate z-scores, while ChCl-LA systems consistently exhibited negative z-scores, indicating lower overall performance. However, it has to be noted that the z-score is a normalization method that assists with the visualization of trends across the obtained data. The obtained values do not indicate statistically significant differences but, rather, patterns relevant to the tested samples.

In comparison to the literature, a study investigating the antioxidant and biological properties of *B.mauritanica* reported that the extract exhibiting the highest antioxidant potential contained saponins, high levels of caffeic acid, and moderate amounts of kaempferol, further highlighting the contribution of phenolic compounds to the antioxidant activity. These constituents were also found to exhibit AChE inhibitory activity, either alone or in combination, indicating the possibility of a synergy among them [4]. As discussed in a recent comprehensive review, the phenolic and flavonoid compounds of *M. comosum* extracts are believed to contribute to its potential roles in regulating lipid metabolism and neurotransmission [29]. Similarly, Casacchia et al. [6] identified two classes of homoisoflavanones, namely, 3 benzyl-4-chromones and the scillascillin type. The 3-benzyl-4-chromone type was found to be the one contributing to the biological activity of the bulb extracts. These compounds were associated not only with strong antioxidant capacity but also with significant lipase inhibition, suggesting that the enzyme-modulating potential of *M. comosum* is connected to its homoisoflavonoid content.

### 2.5. Identified Phytochemicals in the Prepared Extracts Using UHPLC-q-Orbitrap-MS

**Figure 3:**
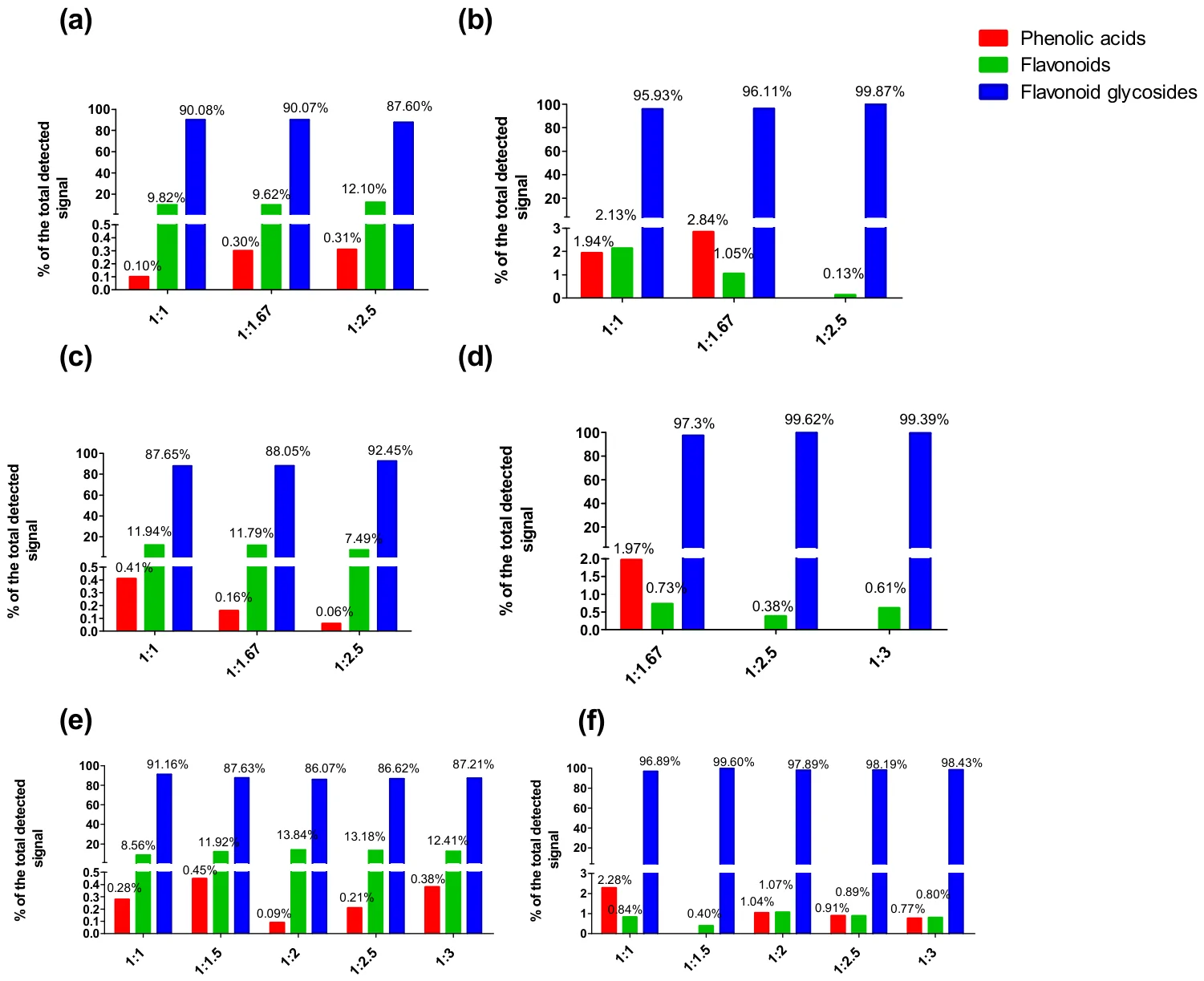
Relative percentage (%) of the detected MS signals of phenolic acids, flavonoids and flavonoid glycosides obtained from (**a**) LA-based extracts, fraction 1, (**b**) LA-based extracts, fraction 2, (**c**) CA-based extracts, fraction 1, (**d**) CA-based extracts, fraction 2, (**e**) MA-based NADES, fraction 1, and (**f**) MA-based extracts, fraction 2. In all cases, 1 mM ChCl was used as HBA, and the extracts concentration was 10 mg/mL. The percentages represent relative MS signal intensities and should not be interpreted as quantitative concentrations of the analytes. The three analyte groups were composed of the following compounds; phenolic acids: protocatechuic acid, p-coumaric acid, ferulic acid, caffeic acid, vanillic acid, chlorogenic-acid isomer, and quinic acid; flavonoids: luteolin, apigenin, acacetin, quercetin, kaempferol, genistein, naringenin, and eriodictyol; and flavonoid glycosides: isoquercetin, hyperoside, vicenin-2, vitexin, orientin, schaftoside, and luteolin diglycoside.

A total of 21 compounds (out of 37) were qualitatively identified in *B. dubia* flower extracts and level 1 identification was achieved for 10 of them (Tables S2 and S3). The profile of bioactive compounds detected and annotated in both NADES and conventional extractants was similar. Particularly, flavonoid glycosides represent the dominant class of compounds detected both in MeOH and NADES systems (Figure S2 and Figure 3, respectively). In the case of NADES, higher relative MS signals for these compounds were observed in the second fraction (Figure 3b,d,f). On the other hand, phenolic acids exhibited higher relative MS signals in the aqueous extract than in the methanolic extract (Figure S2), which may be related to their high polarity (Figure S2). A higher total signal was noticed for flavonoids in water extracts. This is surprising because water–methanol (50–80% of MeOH) mixtures have been proven more efficient in extracting flavonoids as water swells the plant material, increasing surface area, while methanol dissolves the compounds, leading to higher yields [30]. Nevertheless, only qualitative analysis was performed within this study, and such signal differences may be related to the ionization efficiency of each extractant, resulting in different responses as well as solvent-specific matrix effects. Additionally, certain compounds were detected in specific NADES extracts but not in the conventional extracts. *p*-Coumaric acid was found only in ChCl-LA and ChCl-MA systems (1:1 and 1:3, respectively), and kaempferol in the ChCl-MA 1:2.5 of fraction 1 and in most of the extracts of fraction 2. Furthermore, hyperoside, and a chlorogenic acid isomer, were detected only in the NADES extracts.

**Figure 4:**
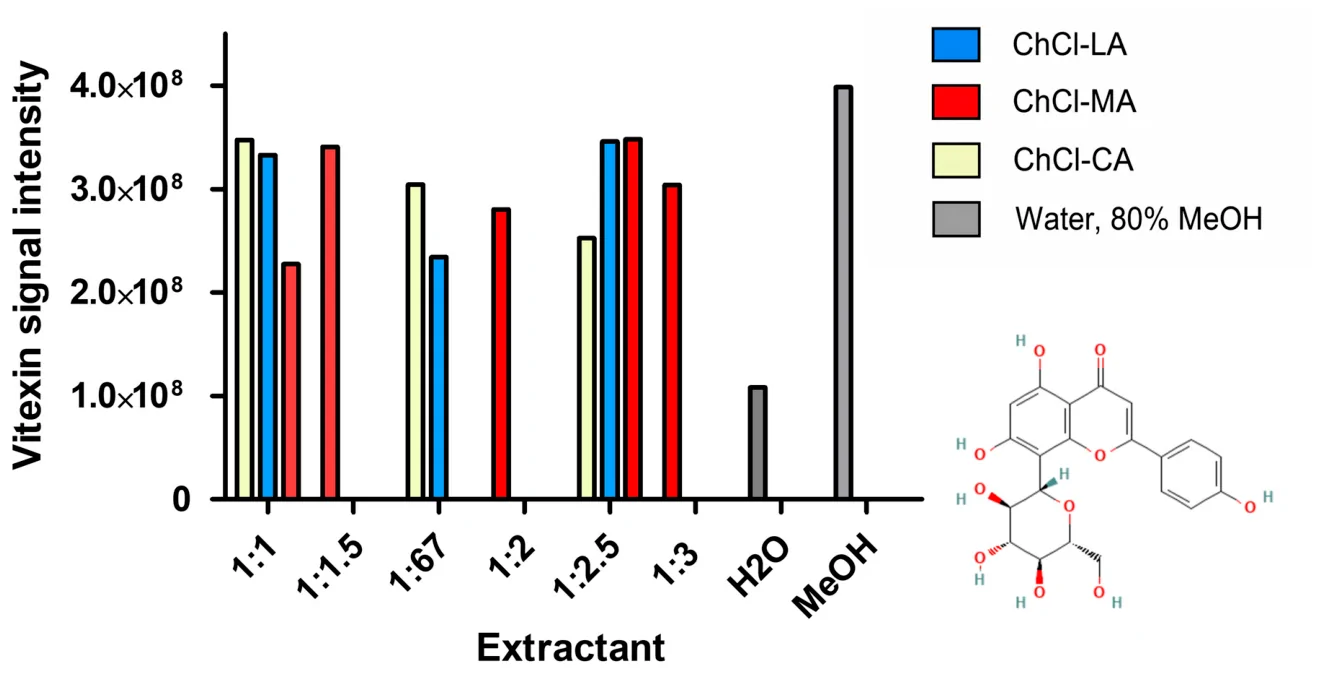
Effect of extractant composition and molar ratio on vitexin-signal intensity.

Vitexin was found in extracts obtained by using both NADES and conventional solvents, with high peak areas. This might be due to vitexin’s low logP = 0.2 indicating a highly polar character, which explains its occurrence in the mentioned extracts. As shown in Figure 4, a higher relative MS signal intensity for vitexin was observed in the NADES extracts compared with the aqueous extract. Regarding the NADES systems, ChCl-CA 1:1 and ChCl-MA 1:2.5 had the highest signal. Depending on the molar ratio between ChCl and organic acid, we can identify two cases. When this ratio is set to 1:1, the highest signal was obtained for the most polar CA system. Nevertheless, when the molar ratio between ChCl and organic acid was set to 1:2.5, the opposite trend was observed, with the less polar MA-system producing the highest signal, followed by the LA system (in close proximity). It is worth mentioning that the findings addressed here are just qualitative estimations related to the total recorded signal, and they do not represent quantitative results. Additionally, the vitexin signal intensity might also be influenced by the injection solvent since NADES were reconstituted in the mobile phase while MeOH and aqueous extracts were directly injected. To confirm these findings, quantitative analysis is necessary upon successful method validation, which falls outside the scope of this study.

Furthermore, the chlorogenic acid isomer and luteolin diglycoside were initially assigned to chlorogenic acid and rutin, respectively, based on their mass spectral characteristics and retention times. In the case of chlorogenic acid, the signal was quite weak, but the retention time did not match the standard. A comparison with the literature indicated that these compounds were more consistent with chlorogenic acid isomers and luteolin diglycoside, respectively [31,32,33]. This suggests that analyte identification should be performed cautiously and always by following strict identification criteria.

Overall, our findings are consistent with previous reports highlighting the plant’s high flavonoid content and antioxidant properties [3,4]. Notably, Jaiswal and Lee [29] compiled a list of 85 bioactive compounds, primarily flavonoids and homoisoflavones. Several of these compounds overlap with those identified in the present study, further supporting the chemical similarity between the *Muscari* and *Bellevalia* genera. While other studies have emphasized the plant’s high phenolic acid content [34], our results show that flavonoids were the primary compounds. The chemical composition depends not only on the polarity of the extractants but also on the genus, as it can be an inherent characteristic of *Bellevalia*. Therefore, differences observed between studies may also be attributed to the investigation of different *Bellevalia* species.

Flavonoids and their derivatives have been widely investigated as potential PNLIP inhibitors, generally showing promising activity [35]. Their inhibitory potential appears to be strongly structure-dependent, as specific features of the flavonoid backbone influence their binding interactions with PNLIP. Structural characteristics such as the C2=C3 double bond, hydroxylation pattern, pyrogallol groups on the B-ring, and the presence of alkyl groups have been reported to affect enzyme inhibition and binding affinity [36]. Previous studies have reported that flavones may exhibit stronger PNLIP inhibitory activity compared to other flavonoid subclasses. While glycosides may not be as potent as their aglycones, they can act as carriers for their aglycones in vivo, thereby improving solubility. In particular, after deglycosylation, the aglycones can bind directly to digestive enzymes, such as PNLIP, in the lumen. Thus, although glycosylation may reduce direct binding affinity, it may positively contribute by enhancing solubility, stability, and bioavailability [37,38].

Similarly, the structural characteristics of both flavonoid aglycones and glycosides also influence AChE inhibitory activity. For instance, docking analysis demonstrated that kaempferol exhibited strong affinity towards the active site of AChE, whereas diglycosides displayed lower inhibitory activity, likely due to the steric hindrance that restricts effective interaction with the enzyme binding site [39]. Overall, flavonoids may exert neuroprotective effects through antioxidant and anti-inflammatory mechanisms. Their ability to stabilize cholinergic neurotransmission and reduce neuroinflammation could play a potential role in treatment strategies for neurodegenerative diseases [40].

### 2.6. Exploratory Data Analysis Based on PCA

**Figure 5:**
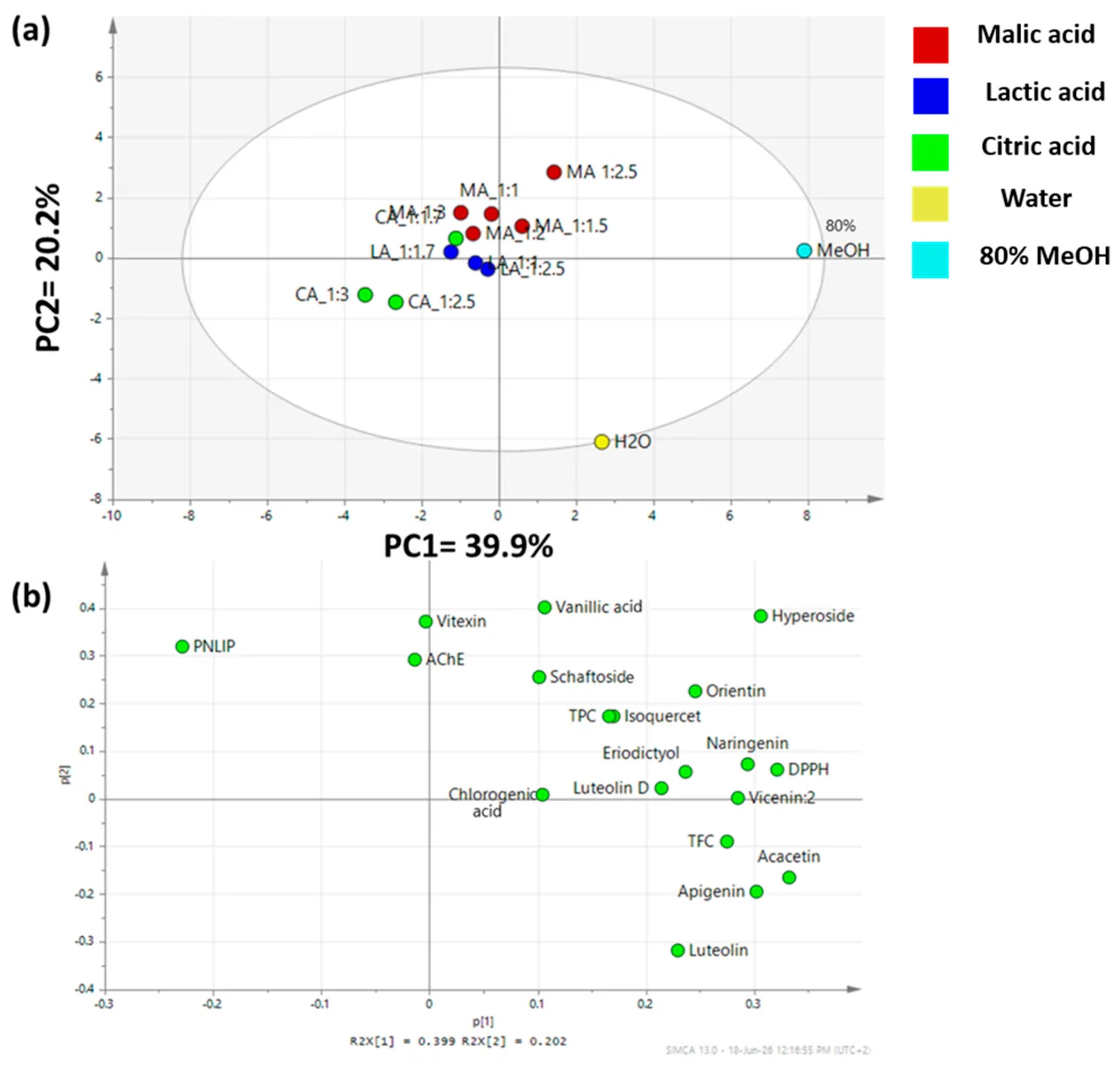
PCA of *B. dubia* extracts based on phenolic composition and assay results: (**a**) score plot showing the distribution of samples according to extraction solvent and (**b**) loading plot illustrating the contribution of phenolic compounds, chemical assays and enzyme-inhibitory activities. The concentration of all tested extracts was 10 mg/mL.

Principal component analysis (PCA) was performed to examine the relationships among extraction systems, phytochemical composition, and enzyme inhibition. The first two principal components explained approximately 60% of the spectral variance (PC1 corresponded to 39.9% and PC2 to 20.2%), while the first five principal components explained about the 89% (PC3 = 13.7%, PC4 = 8.2% and PC5 = 6.3%). The score plot presented a clear separation between conventional solvents and NADES extracts (Figure 5). Methanol (80% MeOH) was positioned on the positive side of PC1, whereas the aqueous extract was distinctly separated toward negative PC2 values, revealing pronounced differences in phytochemical composition and bioactivity profiles. In contrast, NADES extracts formed a compact cluster, reflecting greater similarity among these extraction systems. Within the NADES group, differentiation was primarily observed along the PC2 axis. ChCl-MA extracts were consistently distributed in the upper region of the score plot, exhibiting positive PC2 values. In contrast, ChCl-CA extracts at molar ratios of 1:3 and 1:2.5 were mainly located in the lower-left quadrant, corresponding to negative PC1 and PC2 values. ChCl-LA extracts occupied an intermediate position (Figure 5a).

Among all NADES extracts, the ChCl-MA 1:2.5 system displayed the most distinctive profile, being positioned in the upper-right quadrant of the score plot. This location is consistent with the direction of AChE inhibition, vitexin, hyperoside, vanillic acid, and schaftoside in the loading plot, showing a closer association with these compounds and bioactivities. Its positive PC1 values indicate that differentiation is related to both AChE inhibition and the presence of several bioactive phenolic constituents. Overall, the PCA results demonstrate that both the type of HBD and the choline chloride-to-organic acid molar ratio influenced the extraction profile.

The loading plot showed that PC1 was mainly linked to several bioactive compounds, such as hyperoside, orientin, isoquercetin, eriodictyol, naringenin, vicenin-2, apigenin, acacetin, and luteolin, as well as to TPC, TFC, and DPPH activity. TPC was positioned close to isoquercetin and orientin, indicating a positive association between them. DPPH was located close to naringenin and vicenin-2 in the PCA, suggesting a possible association between these compounds and antioxidant activity. PC2 was mostly influenced by AChE inhibition and compounds with high positive PC2 loadings, especially vitexin, hyperoside, vanillic acid, and schaftoside. The closeness of AChE and vitexin suggests an association between this flavonoid and AChE inhibition (Figure 5b).

Recent reviews have highlighted the potential therapeutic value of vitexin in neurodegenerative disorders, focusing on its role in oxidative stress, neuroinflammation, abnormal protein aggregation and cognitive impairment [41,42]. Experimental studies further support these observations. Malar et al. [43] reported that vitexin is able to protect neuronal cells from oxidative stress by reducing reactive oxygen species production, enhancing antioxidant defenses and preserving mitochondrial function. In an in vivo study, vitexin improved memory retrieval and reversed scopolamine-induced cognitive impairment in rats, suggesting it benefits cognitive function via cholinergic signaling modulation [44]. Moreover, molecular-docking studies have highlighted vitexin as a promising AChE inhibitor. Two molecular-docking studies suggested that vitexin may act as an acetylcholinesterase inhibitor. While Akili et al. [45] identified vitexin as one of the most promising compounds for AChE inhibition, Karaoğlan and Koca [46] further explained this activity by demonstrating favorable interactions between the enzyme active site and the benzopyran ring and hydroxyl groups of the molecule. These observations support the potential contribution of vitexin to the AChE inhibitory activity, highlighting the need for further investigation.

In addition to vitexin, other phenolic compounds identified in the extracts have also been associated with relevant biological effects. A review on the medicinal properties of orientin highlighted its antioxidant and neuroprotective activities [47]. Yu et al. [48] showed that orientin improved cognitive performance in Alzheimer’s disease mouse models and significantly reduced oxidative stress markers, including reactive oxygen species (ROS). It should be noted that, although PCA can reveal potential correlations between chemical compounds and biological activities, these associations should be interpreted with caution. The identification of direct relationships between specific phenolic compounds and bioactivities remains complex, as biological activity often results from the combined effects of multiple metabolites.

### 2.7. Selection of the Optimal NADES

The results of the chemical and enzyme assays generated the following predicted solutions (Table 2), which are ranked according to their overall desirability score. The optimum combination (desirability = 0.832) corresponded to ChCl-MA at a molar ratio of 1:2.47, based on the desirability analysis, which was very close to the experimentally tested ratio of 1:2.5, indicating that this system effectively enhanced the solubilization of phenolic and flavonoid compounds while maintaining strong enzyme-inhibition activities. This observation is in agreement with the experimental results, where 1:2.5 ChCl-MA extracts exhibited a distinct profile.

Several reports have indicated that eutectic mixtures containing MA exhibit a balanced combination of polarity and viscosity, which supports the effective solubilization and diffusion of phenolic compounds through plant tissues. Gómez-Urios et al. [49] showed that ChCl-MA systems were effective for extracting bioactive compounds from orange peel, while also enhancing their stability and antioxidant capacity. In a recent study, the ChCl-MA system was identified as one of the most effective solvents, among a series of NADES, for the extraction of phenolic compounds from *Carya cathayensis* Sarg. peels (Chinese hickory) [50]. Furthermore, ChCl-MA was reported as a highly promising extraction solvent for anthocyanin recovery from jaboticaba peel, while also exhibiting a strong protective effect against thermal degradation of the extracted compounds [51]. Compared with the more viscous ChCl-CA systems, the hydrogen-bonding structure of ChCl-MA can promote molecular mobility, which could explain the higher phenolic yields recorded in the present work. However, the selection of the optimal solvent depends not only on its physicochemical properties, but also on the nature of the matrix and the target compounds [12].

## 3. Materials and Methods

### 3.1. Chemicals

All solvents, standards, and analytical reagents used in this study were of analytical or HPLC grade. MeOH (≥99.8%), LA (analytical reagent grade, ≥90.1%), and CA monohydrate were purchased from Fisher Chemical (Loughborough, UK). DL-MA, ChCl, and formic acid (FA, ≥99.9%, analytical grade) were obtained from Sigma-Aldrich (St. Louis, MO, USA). Gallic acid (≥99.5%, research grade) was supplied by Serva Feinbiochemica (Heidelberg, Germany), and the Folin–Ciocalteu reagent was purchased from Carlo Erba Reagents (Cornaredo, Italy).

Additional reagents and analytical standards used for the antioxidant activity and TPC and TFC assays, quercetin (≥95%), and 2,2-diphenyl-1-picrylhydrazyl (analytical standard), were all obtained from Sigma-Aldrich (St. Louis, USA). The hydrochloric acid solution (37%) and aluminum chloride (≥98%) were purchased from Merck (Darmstadt, Germany).

Phosphate buffer saline (PBS) tablets, dimethyl sulfoxide (DMSO, purity 99%), and ethanol (EtOH, purity 99%), used as a buffer and reagents, respectively, in the assays, were supplied by Sigma Aldrich (Prague, Czech Republic). All 96-microwell-plate assays were performed in plates purchased from Gama Group (Ceske Budejovice, Czech Republic). AChE from *Electrophorus electricus*, acetylthiocholine iodide (AThI purity > 99%), 5,5-dithiobis-2-nitrobenzoic acid (DTNB, purity > 99%) and huperzine-A (purity > 98%) were purchased from Sigma Aldrich (Prague, Czech Republic) and used for the AChE assay. Type II porcine PNLIP, p-nitrophenyl acetate (NPA, purity > 98%) and orlistat (purity > 98%) were bought from Sigma Aldrich (Prague, Czech Republic) and were used for the PNLIP assay.

### 3.2. Samples and Sample Preparation

The plant material was purchased from a local market on the island of Zakynthos in western Greece in March 2022. Upon transportation to the lab, the plant material was botanically identified by a plant taxonomist, and this contribution is gratefully acknowledged. The flowers were separated from the stems, placed in trays at −20 °C for 24 h and freeze-dried (Unicryo MC 2 L −60 °C, GmbH, Munich, Germany). The dried material was powdered using a grinder (Janke and Kunkel IKA-WERKE analytical mill, Staufen, Germany) and stored in airtight containers at −20 °C for further use.

### 3.3. Preparation of NADES and Experimental Design

The ΝΑDES were prepared according to a previously reported protocol [28], with slight modifications. In brief, ChCl was combined with different HBDs (LA, MA and CA) in a flask according to the molar ratio shown in Table 3. The required masses of ChCl and each HBD were calculated based on their respective molecular weights and the target molar ratio. The mixtures were placed in a rotary evaporator and heated at 80 ± 5 °C with continuous mixing until complete melting was achieved. Then, each prepared mixture was weighed, and distilled water corresponding to 30% of the mass of the NADES was added. The resulting mixtures were transferred to a heating plate and stirred at 50 °C until a homogeneous solution was obtained. Although the term “eutectic” can only apply to one specific ratio of HBA and HBD, the precise term for the solvents used in the present study is “low-melting mixtures”. However, due to widespread use of the term “NADES” in the literature, the term is still used for the studied solvents.

Optimization of the extraction process with respect to molar ratio and the number of carboxylic groups was achieved by implementing an I-optimal design (point exchange method, quadratic model) using Design-Expert^®^ software (Version 11, Stat-Ease Inc., Minneapolis, MN, USA). The design was constructed using two independent variables. Detailed information on the models, their statistical parameters, and the criteria used for model selection is provided in the Supplementary Materials (Table S4).

A: The molar ratio of the ChCl:HBD (e.g., ChCl:1, ChCl:2).

B: The number of carboxylic groups as a categoric factor (level 1 for lactic acid, level 2 for malic acid, and level 3 for citric acid).

TPC, TFC, antioxidant activity, and enzyme inhibition against PNLIP and AChE were selected as responses. Seventeen runs were generated (Table 3) to ensure a balanced distribution of experimental points and robust estimation of model parameters. Repeated runs represented independent extraction replicates, as a separate extraction was performed in each case, including those with identical experimental conditions. The overall desirability was calculated using Design-Expert^®^ software (Version 11, Stat-Ease Inc., Minneapolis, MN, USA) to identify the most efficient combination of ΝΑDES, where 1.0 represents the most desirable combination for all the responses. The desirability method uses an objective function, and reflects the desirable ranges for each response. More information about the function can be found here: https://www.statease.com/docs/v23.1/contents/optimization/desirability-details/ (accessed on 10 September 2026).

### 3.4. Ultrasound-Assisted Extraction (UAE)

The UAE was carried out according to previously reported studies, with slight modifications [25,52]. First, 2 g of the dried *B. dubia*-flower powder was mixed with 2 mL of the above-prepared NADES (30% water content, *w*/*w*) in 50 mL centrifuge tubes. Then, the mixtures were placed in an ultrasonic bath and sonicated at 40 ± 5 °C for 120 min. Every 30 min, the system was removed from the ultrasonic bath and vortexed for 1 min to enhance the contact between the solvent and the material. At the end of the extraction, 2 mL of deionized water was added to the samples to facilitate the collection of the supernatant. The supernatant was obtained by centrifugation (Rotina 380R, Hettich, Germany) at 10,000 rpm for 10 min. An equivalent extraction procedure was also applied using H_2_O and 80% MeOH as conventional solvents. However, due to their lower viscosity and cleaner extract profile, no post-centrifugation adjustment step was required.

### 3.5. Total Phenolic Content

TPC was determined by a previously reported method, with slight modifications [53]. Specifically, 40 μL of the extracts were mixed with 160 μL of 0.5% (*v*/*v*) formic acid, followed by the addition of 0.8 mL of distilled water and 5 mL of Folin–Ciocalteu reagent (1:10, *v*/*v*). After 3 min, 4 mL of sodium carbonate (Na_2_CO_3_, Penta, Prague, Czech Republic) solution (7.5% *w*/*v*) was added, and the content was vortexed. For the blank, 200 μL of diluted solvent, 1:50 (*v*/*v*) with formic acid was added instead of the extract. After 1 h of reaction in the dark, at room temperature (around 25 °C), the absorbance was measured at 765 nm using a UV–Vis double-beam spectrophotometer (Jasco V-530, Tokyo, Japan). A calibration curve was made using gallic acid (10–80 μg/mL) prepared in H_2_O. The results were expressed as milligram equivalents of gallic acid per gram of dried plant material (mg GAE/g of dried plant material). The same protocol was also applied for the methanolic (80% MeOH) and aqueous extracts, except that the dilution step was performed using distilled water instead of formic acid, as these solvent systems exhibited lower viscosity and adequate clarity, eliminating the need for acid adjustment.

### 3.6. Total Flavonoid Content

The aluminum chloride method was applied, according to Wairata et al. [54]. A total of 500 μL volume of each extract was diluted 1:10 with MeOH and mixed with 500 μL of 2% AlCl_3_ (in MeOH). After 1 h of reaction in the dark, at room temperature (around 25 °C), the absorbance was measured at 415 nm using a UV–Vis double-beam spectrophotometer (Jasco V-530, Tokyo, Japan), with methanol as the blank. The total content of flavonoids was determined using quercetin in methanol as a standard (0–50 μg/mL). The total flavonoid content was expressed as milligram equivalents of quercetin per gram of dried plant material (mg QE/g dried plant material). The same analytical procedure was applied to the methanolic (80% MeOH) and aqueous extracts, except that the dilution factor increased to 1:20, to achieve comparable absorbance values within the linear range of the calibration curve.

### 3.7. Determination of Antioxidant-Activity Assay

The antioxidant activity of the flower extracts was determined following a previously reported paper [55]. The extracts were diluted 1:2 (*v*/*v*) with water to a final volume of 200 μL, followed by the addition of 1.8 mL of methanol and 2 mL of freshly prepared DPPH solution (in MeOH). The content was vortexed and after 30 min of reaction in the dark, at room temperature, the absorbance was measured at 515 nm using a UV–Vis double-beam spectrophotometer (Jasco V-530, Tokyo, Japan). The antioxidant activity was determined using gallic acid in methanol as a standard (0.4–4 mg/mL). For the control, a mixture of 100 μL of each NADES and 100 μL of distilled water was used instead of the extract to reproduce the matrix effect of the solvent system. Results were expressed as milligram equivalents of gallic acid per gram of dried plant material (mg GAE/g dried plant material).

The DPPH scavenging activity was also calculated as percentage inhibition, expressed as radical scavenging activity (RSA), using the following equation.

%RSA = [(A_control_ − A_extract_)/A_control_] × 100

The same analytical procedure was applied for the methanolic (80% MeOH) and aqueous extracts, except that the dilution ratios were adjusted to ensure absorbance values within the linear range of the assay.

### 3.8. Measurement of the Color Stability of NADES Extracts

The redness index (a*) from the CIE Lab* color space was selected as the primary color parameter, as it is highly sensitive to pigment degradation and hue shifts toward brownish tones. The color of the extracts was measured using a Lovibond Spectrocolorimeter LC100 (The Tintometer Ltd, Dortmund, Germany). The extracts were stored under refrigerated conditions (approximately 4 °C), and color stability was monitored at 4-day intervals over a period of one month. For each extract and time point (t), the measured value was recorded as a_t_, while the initial value at t = 0 was denoted a_0_.

The degradation of the red color component was analyzed through kinetic modeling of a* over time. Two linearized models were tested:

- Zero-order kinetics:

$$a_{t}=a_{0}-k_{0}t$$

- First-order kinetics:

$$ln(a_{t})=ln(a_{0})-k_{1}t$$

where a_t_ is the redness index at time t, and k_0_ and k_1_ represent the degradation rate constants for zero- and first-order reactions, respectively. Both zero- and first-order kinetic models were fitted to the experimental data. The model with the higher R^2^ was considered to provide the better description of the experimental data, while R^2^ > 0.90 was used as a minimum criterion to avoid assigning a kinetic model to poorly fitted data [25,56]. Systems for which neither model reached this criterion were not assigned to a kinetic model and were excluded from the corresponding kinetic interpretation. From the selected model, the following color stability parameters were derived:

Half-life (t_1_/_2_): the time required for the a* value to decrease to half its initial value.

- Zero-order:

$$t_{1/2}=\frac{a_{0}}{2k_{0}}$$

- First-order:

$$t_{1/2}=\frac{ln(2)}{k_{1}}$$

Destruction time (D_90_): the time corresponding to a 90% reduction in a*.

- Zero-order:

$$D_{90}=\frac{0.9a_{0}}{k_{0}}$$

- First-order:

$$D_{90}=\frac{ln(10)}{k_{1}}$$

### 3.9. In Vitro Enzyme-Inhibition Assays

#### 3.9.1. AChE Assay

To monitor the inhibitory effect of the extracts on AChE, an in-house AChE assay was used, with slight modifications [57]. Firstly, 60 uL of AChE (0.009 U per well) was incubated with 20 uL of a sample for 15 min. Next, 20 uL of a solution of AThI (5 mM) and DTNB (1.5 mM) in a 9:1 ratio (both prepared in PBS) were added and the absorbance was measured at 412 nm after 2 min. A solution of huperzine A (50 mg/L), a known AChE inhibitor, was used as the positive control, whilst PBS was used as the negative control. Sample blanks were also prepared, and PBS was used instead of the AChE solution to subtract the absorbance not related to the enzyme catalytic activity. For each sample, all measurements were performed in triplicate and the results are expressed as mean ± standard deviation.

#### 3.9.2. PNLIP Assay

The inhibitory effect of the tested extracts on PNLIP was monitored using an in-house assay [57]. Briefly, a PNLIP solution (1250 μg/mL) was prepared daily in PBS. Solution centrifugation was necessary to eliminate insoluble impurities contained in the dried PNLIP powder (10,000 rounds per minute at room temperature for 2 min; Rotina 380R, Hettich, Germany). Next, 80 μL PNLIP was incubated with 10 μL of extract for 15 min. Then, 10 μL of 10 mM NPA in EtOH were added and the absorbance was measured at 405 nm after 15 min. For each sample, all measurements were performed in triplicate. Orlistat (50 μg/mL) was used as a positive control. Sample blanks were also prepared, and PBS was used instead of the PNLIP solution to subtract the absorbance not related to the enzyme catalytic activity.

For all enzyme assays, methanolic (80% MeOH) and aqueous (H_2_O) extracts were also analyzed to assess potential differences. Values are expressed as the mean of three replicates ± standard deviation.

### 3.10. UHPLC-q-Orbitrap-MS Analysis to Identify Bioactive Compounds Through Suspect Screening

Phenolic compounds (Fraction 1) and anthocyanins (Fraction 2) were purified using a solid-phase extraction (SPE) procedure, as previously described [58]. Before loading the sample, the C18 SPE cartridge (500 mg, Macherey-Nagel, Düren, Germany) was activated with 6 mL of acidified methanol (0.01% *v*/*v* HCl), followed by 6 mL of acidified distilled water (0.01% *v*/*v* HCl). Five milliliters of a diluted sample (1:50 with deionized water) were loaded onto the cartridge, which was subsequently washed with acidified distilled water (12 mL) to remove non-retained compounds such as sugars and organic acids. The sorbent was dried under a nitrogen stream, and phenolic compounds were eluted with ethyl acetate (12 mL). The anthocyanins were eluted by rinsing the cartridge with acidified methanol until complete discoloration of the sorbent was observed. The two fractions were concentrated with nitrogen stream close to dryness and reconstituted in the initial mobile phase composition of the chromatographic analysis (final extract concentration was 10 mg/mL), stored in vials and kept at −20 °C until analysis. Additionally, aqueous and methanolic extracts prepared as described in Section 3.4 were also chromatographically analyzed (final extract concentration was 10 mg/mL).

UHPLC-q-Orbitrap-MS analysis was performed on a Vanquish Horizon chromatograph coupled with an Exploris 240 mass spectrometer (Thermo Fisher Scientific, Waltham, MA, USA). The following parameters were set: ion transfer-tube temperature and vaporizer temperature were 250 °C; capillary voltage was 4000 V; and sheat, aux and sweep gas were 45, 10 and 0 (arb). The acquisition mode was programmed to obtain spectra in full MS mode and to obtain MS/MS spectra. The parameters of full scan and data-dependent MS2 (ddMS^2^) experiments were as follows: Orbitrap resolution 60,000 (MS1) and 15,000 (MS2); scan range 100–1200 *m*/*z*; isolation window 1 *m*/*z*; normalized HCD collision energy (%) 15 and 35; and 20 dependent scans. The Thermo Scientific Xcalibur software version 4.3 was used to generate the total ion chromatograms. To control the instrument, the Xcalibur and Orbitrap Exploris Tune (version 4.4, Thermo Fisher Scientific, Waltham, MA, USA) software was utilized. The separation was carried out in an Acquity UPLC HSS T3 (2.1 × 100 mm, 1.8 µm; Waters, Milford, MA, USA) analytical column at 45 °C. The mobile phase consisted of A: deionized water with 0.1% formic acid and B: methanol with 0.1% formic acid. The gradient used was: 0–1 min (5% B), 1–8 min (5–100% B), 8–15 min (100% B), and 15–17 min (5% B). The injection volume was 2 µL and the flow rate was 0.4 mL min^−1^. Mass spectra were obtained both in negative and positive ionization mode.

A suspect-screening approach was implemented to identify bioactive compounds in the prepared extracts. The initial suspect list contained 37 phytochemicals already reported in *B. dubia* [3,4]. The identification was performed based on the exact mass, mass error (<5 ppm), isotope profile and conformity of the mass fragmentation spectra with online databases (such as www.mzcloud.org, www.pubchem.com, www.metlin.com, and https://massbank.eu; accessed on 7 August 2026). Identification levels from 1 to 3 were achieved (see Section 2.5). Level 1 corresponded to a confirmed structure by comparison to an analytical standard, including matching accurate mass, MS/MS spectrum, and retention time measured under the same analytical conditions. In our case, 16 analytical standards were available; namely, protocatechuic acid, *p*-coumaric acid, ferulic acid, sinapic acid, chlorogenic acid, caffeic acid, quinic acid, luteolin, isoquercetin, kaempferol, genistein, daidzein, quercetin, catechin, naringenin and rutin. All standards were of analytical grade and purchased from Sigma Aldrich (Prague, Czech Republic). In level 2, an exact chemical structure could be proposed with high confidence based on strong evidence such as a library spectrum match or diagnostic MS/MS evidence, but it was not confirmed with a reference standard. A level 3 identification corresponded to a tentative candidate(s), since the acquired data supported one or several possible structures. More information on the identification levels can be found in Schymanski et al. [59,60].

### 3.11. Applied Statistical Analysis and Chemometrics

To critically compare the results acquired across the different extraction systems for all the tested assays, the z-score was calculated. A z-score measures how many standard deviations a specific data point is from the respective mean value. To calculate it, the following formula was used:

z = (x − μ)/σ

where x = the original value, μ = mean of that variable and σ = standard deviation of that variable. If a z-score is positive, it indicates that the data point is above the mean, while, if negative, it indicates that it is below the mean.

The non-supervised PCA was used to uncover potential trends and clusters by fusing the in-house assay results with the chromatographic findings obtained by the extracts. For this purpose, the two fractions of SPE were combined (in each case, the area of a certain compound in fraction 1 and 2 was summed) as they had the same concentration (10 mg/mL) and were originally contained in the same crude extract. To process the data, the attained assay responses as well as chromatographic peak areas were exported to a MS Excel 2019 file and imported into the SIMCA package (version 13.0.3.0; Gottingen, Germany). The raw chromatographic data were first logarithmically transformed to stabilize variance across a wide dynamic range. Then, both chromatographic and assay data were autoscaled to ensure that all variables have an equal influence on the model, regardless of their original magnitude.

## 4. Conclusions

The optimal NADES based on the desirability analysis was ChCl-MA at a molar ratio of 1:2.47 (experimentally 1:2.5), indicating that this system effectively achieved high yields of TPC, TFC, and antioxidant activity, while also exhibiting strong enzyme inhibition. The NADES containing CA at a ratio of 1:1.67 exhibited the greatest color stability, as indicated by its kinetic parameters and half-life, possibly due to higher viscosity compared to the other organic acid-based extracts. In terms of extract composition, a total of 21 compounds were detected and annotated, including phenolic acids, flavonoids, and flavonoid glycosides. PCA revealed that ChCl-MA, especially the 1:2.5 system, was clearly clustered away from the rest of the systems, indicating a distinct chemical and bioactive profile. Notably, in the case of this system, vitexin and AChE inhibition were associated, which requires further investigation. Overall, the findings highlight the potential of NADES as alternative extraction media for obtaining bioactive compounds from *B. dubia* and provide a basis for further optimization and investigation of these systems. Future studies should include the characterization of the physicochemical properties of NADES, such as polarity, viscosity, and hydrogen-bonding interactions, to better understand their influence on extraction efficiency. In addition, the TFC assay was performed without analytical replicates, and these results should, therefore, be interpreted with caution. As a next step, quantitative UHPLC-MS analysis is currently underway to provide a more accurate assessment of the identified bioactive compounds and their extraction efficiency. Further work will also focus on evaluating safety, bioavailability, and in vivo efficacy to support the potential practical application of these extracts.

## Figures and Tables

**Table 1:** Half-life (t_1_/_2_) and destruction time (D_90_) of color degradation for *B. dubia* flower extracts obtained with different NADES combinations.

DESs Extracts	Half-Life (Day)	Destruction Time (Day)
ChCl-LA 1:1	21	37
ChCl-LA 1:2.5	38	68
ChCl-MA 1:1	40	133
ChCl-MA 1:1.5	14	47
ChCl-MA 1:2	38	125
ChCl-MA 1:2.5	24	44
ChCl-MA 1:3	23	77
ChCl-CA 1:1.67	51	169

**Table 2:** Suggested solutions and desirability using Design-Expert software.

Suggested NADES System	Molar Ratio of the Organic Acid	Organic Acid	TPC (mg GA/g ^1^)	DPPH (mg GA/g)	TFC (mg QE/g)	AChE (% Inhibition)	PNLIP (% Inhibition)	Desirability
1	2.47	MA	15.69	0.65	10.72	78.21	76.66	0.83
2	1.00	CA	14.29	0.53	10.26	36.60	78.78	0.65
3	3.00	LA	9.40	0.39	6.30	21.81	48.34	0.25
4	1.00	LA	12.23	0.14	6.82	9.39	56.64	0.23
5	3.00	CA	11.46	0.13	9.74	4.98	70.47	0.20

^1^ g dried plant material.

**Table 3:** Experimental design using Design-Expert software.

ChCl as HBA	Molar Ratio of the HBD ^1^	Organic Acids Used as HBD
1	1	CA
1	3	MA
1	2.5	LA
1	1	MA
1	1.5	MA
1	2.5	CA
1	3	CA
1	1	LA
1	2.5	LA
1	2.5	LA
1	2.5	MA
1	2	MA
1	2	MA
1	1	LA
1	1.66667	CA
1	1.66667	CA
1	1.66667	LA

^1^ ChCl as the HBA is constant at 1: HBD.

## Data Availability

The data will be available upon a reasonable request.

## Supplementary Materials

The following supporting information can be downloaded at: https://www.mdpi.com/article/10.3390/molecules31183267/s1, Table S1. Phenolic, flavonoid antioxidant contents, and RSA (%) in *B. dubia* extracts obtained with different ΝΑDES and conventional solvents. Table S2. Level 1 identified compounds in *B. dubia* flower extracts. Table S3. Tentatively identified compounds in the extracts. Table S4. Model adequacy and statistical parameters for the fitted responses. Figure S1. Heatmap depicting the mean values (n = 3 in each case) of the percentage inhibition of the two key enzymes, AChE and PNLIP, by *B. dubia* flower extracts prepared using different NADES. Figure S2. Percentage of the total detected signal of phenolic acids, flavonoids and flavonoid glycosides obtained from aqueous and methanolic extracts.
