# Supplementary material for: Experimental Design-Driven Optimization of Choline-Based Natural Deep Eutectic Solvents for the Extraction of Bioactive Compounds from Bellevalia dubia
Source: Molecules. 2026 Sep 15;31(18):3267. doi: 10.3390/molecules31183267 (PMC13609868; doi:10.3390/molecules31183267)
Supplement: Supplementary file 1 [file molecules-31-03267-s001.zip › molecules-4519164-supplementary.pdf]

SUPPLEMENTARY MATERIALS

Experimental Design-Driven Optimization of Choline-Based Natural Deep Eutectic Solvents for the Extraction of Bioactive Compounds from *Bellevalia Dubia*

Anastasia Charalampidopoulou[1,2], Marie Zlechovcova[2], Charalampos Proestos[3], Chrysavgi Gardeli[1*], Aristeidis S. Tsagkaris[2*]

1: Laboratory of Food Chemistry and Analysis, Department of Food Science and Human Nutrition, Agricultural University of Athens, Iera Odos 75, Athens 11855, Greece

2: Department of Food Analysis and Nutrition, Faculty of Food and Biochemical Technology, University of Chemistry and Technology, Technická 5, 166 28, Prague, Czech Republic

3: Food Chemistry Laboratory, Department of Chemistry, National and Kapodistrian University of Athens, 15772 Athens, Greece

*contact: tsagkara@vscht.cz, agardeli@aua.gr

Table S1. Phenolic, flavonoid, antioxidant contents, and RSA (%) in *B. dubia* extracts obtained with different NADES and conventional solvents[1].

Extracts	Total Phenolic Content (mg GAE/g)	Total Flavonoid Content (mg QE/g)	Antioxidant Content (mg GAE/g)	RSA (%)	AChE inhibition (%)	PNLIP inhibition (%)
**ChCl-LA (1:1)**	12.29 ± 6.49	7.05 ± 3.43	0.18 ± 0.15	18.03 ± 12.60	7.63 ± 0.46	55.03 ± 16.76
**ChCl-LA (1:1.67)**	7.99 ± 0.14	4.04	0.08 ± 0.01	8.99 ± 0.50	7.33 ± 1.14	66.94 ± 13.57
**ChCl-LA (1:2.5)**	11.17 ± 0.67	7.14 ± 1.67	0.35 ± 0.05	31.54 ± 4.25	10.09 ± 6.18	43.88 ± 6.49
**ChCl-MA (1:1)**	15.58 ± 0.19	8.60	0.99 ± 0.00	64.71 ± 0.44	11.60 ± 4.47	71.77 ± 7.66
**ChCl-MA (1:1.5)**	18.52 ± 0.48	12.23	1.04 ± 0.01	62.35 ± 1.24	26.81 ± 2.39	73.13 ± 10.38
**ChCl-MA (1:2)**	16.97 ± 0.99	11.84 ± 0.28	0.66 ± 0.04	57.49 ± 3.59	41.37 ± 20.87	76.94 ± 8.27
**ChCl-MA (1:2.5)**	17.20 ± 0.02	11.43	0.70 ± 0.01	60.99 ± 0.57	78.21 ± 12.96	86.74 ± 9.75
**ChCl-MA (1:3)**	12.88 ± 0.13	9.13	0.58 ± 0.01	50.49 ± 0.48	50.10 ± 19.23	86.12 ± 8.95
**ChCl-CA (1:1)**	17.68 ± 0.38	12.39	0.58 ± 0.00	42.61 ± 0.09	38.25 ± 16.55	87.42 ± 5.44
**ChCl-CA (1:1.67)**	12.34 ± 0.40	9.42 ± 0.27	0.36 ± 0.06	27.12 ± 4.60	15.66 ± 5.82	77.01 ± 5.87
**ChCl-CA (1:2.5)**	9.45 ± 0.04	9.48	0.20 ± 0.02	15.81 ± 1.48	3.82 ± 1.14	73.95 ± 7.21
**ChCl-CA (1:3)**	12.81 ± 0.05	9.35	0.18 ± 0.00	13.73 ± 0.12	6.53 ± 4.57	58.44 ± 11.20
**H_2O**	13.39 ± 1.63	14.97 ± 1.79	0.77 ± 0.02	68.74 ± 2.16	0	3.54 ± 0.86
**80% MeOH**	16.13 ± 0.33	18.02 ± 0.73	1.52 ± 0.07	54.36 ± 2.63	1.79 ± 2.05	12.85 ± 6.71

[1]TPC and DPPH were measured in duplicate (n = 2 analytical replicates per extract), whereas TFC was determined once per extract (n = 1). However, some NADES compositions appeared more than once in the experimental design and were therefore represented by multiple independently prepared extracts. For these compositions, the mean and SD were calculated using all measurements obtained from the corresponding independent extracts, resulting in a higher total n depending on the number of repetitions in the experimental design.

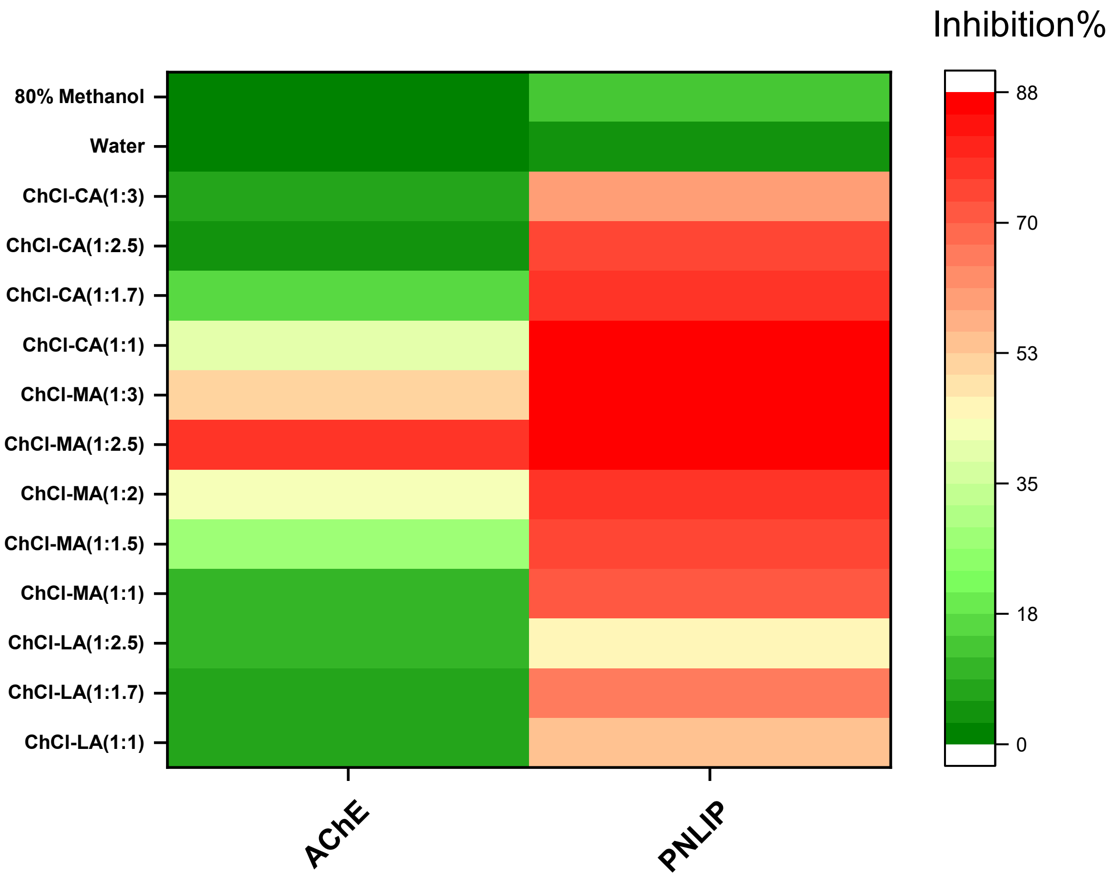

Figure S1. Heatmap depicting the mean values (n = 3 in each case) of the percentage inhibition of the two key enzymes, AChE and PNLIP, by *B. Dubia* flower extracts prepared using different NADES. Water and 80% MeOH were included as controls. The final concentration of all extracts (NADES-based, aqueous, and methanolic) was 0.1 mg/mL in the AChE assay and 0.025 mg/mL in the PNLIP assay.

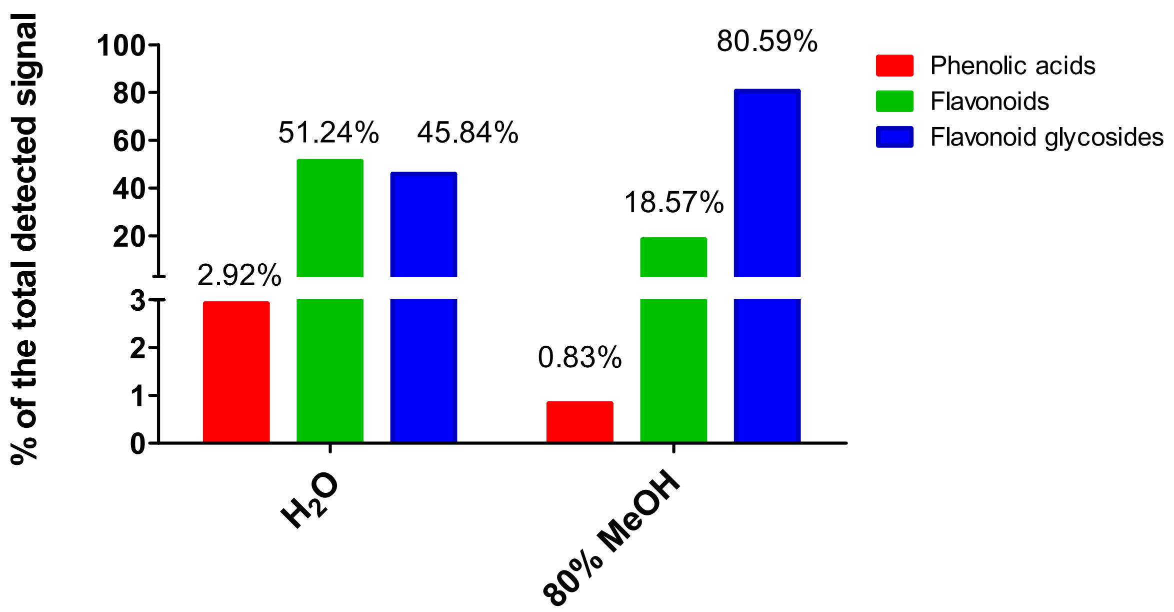

Figure S2. Percentage of the total detected signal of phenolic acids, flavonoids and flavonoid glycosides obtained from aqueous and methanolic extracts. The extract concentration was 10 mg/mL.

Table S2. Level 1 identified compounds in *B. dubia* flower extracts.

Compound	Chemical Formula	Detected ion	Measured *m/z*	Mass error (ppm)	tR (min)	Fragment ions (*m/z*)	Identification level	Contained in	Class
Protocatechuic acid	$C_7H_6O_4$	$[M-H]^-$	153.0194	0.7	2.94	109.0295	1	NADES and conventional solvents	Phenolic acid
Luteolin	$C_{15}H_{10}O_6$	$[M-H]^-$	285.0403	-0.7	6.29	weak MS/MS	1	NADES and conventional solvents	Flavone
p-Coumaric acid	$C_9H_8O_3$	$[M-H]^-$	163.0403	1.2	4.77	weak MS/MS	1	Fr1_ChCl:CA 1:1, Fr1_ChCl:MA 1:3	Phenolic acid
Ferulic acid	$C_{10}H_{10}O_4$	$[M-H]^-$	193.0509	1.6	4.88	117.0348, 149.0610, 193.0504	1	80% MeOH	Phenolic acid
Caffeic acid	C9H8O4	$[M-H]^-$	179.0351	0.6	4.17	135.0452	1	NADES and conventional solvents	Phenolic acid
Quercetin	$C_{15}H_{10}O_7$	$[M-H]^-$	301.0354	0	6.14	151.0037, 178.9986, 121.0294	1	80% MeOH, H2O	Flavonol
Isoquercetin	$C_{21}H_{20}O_{12}$	$[M-H]^-$	463.0872	-2.2	5.29	301.0354, 300.0276	1	NADES and H2O	Flavonoid glycoside
Quinic acid	$C_7H_{12}O_6$	$[M-H]^-$	191.0558	-1.6	0.62	173.0092, 129.0193, 117.0194	1	80% MeOH, H2O	Organic acid
Kaempferol	$C_{15}H_{10}O_6$	$[M-H]^-$	285.0404	-0.4	6.61	227.2551, 162.7851	1	Fr1_ChCl-MA 1:2.5, Fraction 2 and 80% MeOH	Flavonol
Naringenin	$C_{15}H_{12}O_5$	$[M-H]^-$	271.0612	0	6.17	151.0038, 119.0502, 177.0193	1	NADES and conventional solvents	Flavanone

Table S3. Tentatively identified compounds in the extracts.

Compound	Chemical Formula	Detected ion	Measured m/z	Mass error (ppm)	tR (min)	Fragment ions (m/z)	Identification level	Contained in	Class
**Vanillic acid**	$C_8H_8O_4$	[M-H]-	167.0349	-0.5	4.70	152.0114, 108.0218	2	NADES and 80% MeOH	Phenolic acid
**Apigenin/Genistein**	$C_{15}H_{10}O_5$	[M-H]-	269.0456	0.2	6.64	weak MS/MS	3	NADES and conventional solvents	Flavone
**Acacetin**	$C_{16}H_{12}O_5$	[M-H]-	283.0612	0.0	6.84	weak MS/MS	3	NADES and 80% MeOH	Flavone
**Vicenin-2**	$C_{27}H_{30}O_{15}$	[M-H]-	593.1509	-0.5	4.44	383.0771, 353.0664, 473.1089, 503.1195	2	NADES and conventional solvents	Flavone glycoside
**Vitexin**	$C_{21}H_{20}O_{10}$	[M-H]-	431.0977	-1.6	5.15	311.0560, 341.0664, 283.0609	2	NADES and conventional solvents	Flavone glycoside
**Hyperoside**	$C_{21}H_{20}O_{12}$	[M-H]-	463.0883	0.2	5.54	301.0354, 151.0035, 178.9998	2	NADES	Flavonol glyco side
**Eriodictyol**	$C_{15}H_{12}O_6$	[M-H]-	287.0563	0.7	5.31	259.0613, 125.0241	2	NADES and conventional solvents	Flavanone
**Orientin**	$C_{21}H_{20}O_{11}$	[M-H]-	447.093	-0.6	4.83	357.0614, 327.0513	2	NADES and conventional solvents	Flavone glycoside
**Schaftoside**	$C_{26}H_{28}O_{14}$	[M-H]-	563.1401	-0.9	4.78	503.1195, 473.1091, 443.0981, 383.0770, 353.0665	2	NADES and conventional solvents	Flavone glycoside
**3-O-caffeoylquinic acid/4-O-caffeoylquinic acid/5-O-caffeoylquinic acid (chlorogenic acid isomers)**	$C_{16}H_{18}O_9$	[M-H]-	353.0875	-0.9	4.20	weak MS/MS	3	NADES	Phenolic acid
**Luteolin diglycoside**	$C_{27}H_{30}O_{16}$	[M-H]-	609.1456	0.0	4.79	446.0854, 283.0248	3	NADES and conventional solvents	Flavone glycoside

Table S4. Model adequacy and statistical parameters for the fitted responses [1]

Response	Model p-value	Lack of Fit p-value	R^2	Adjusted R^2	Predicted R^2	Model Adequacy
**Total Phenolic Content (mg/g)**	0.0114	0.4825	0.5608	0.4595	0.2065	6.3357
**Antioxidant activity (mg/g)**	<0.0001	0.7866	0.8831	0.8300	0.6945	10.7385
**Total Flavonoid Content (mg/g)**	0.0231	0.8053	0.5072	0.3935	0.1199	4.5325
**AChE Inhibition**	<0.0001	0.8367	0.9803	0.9607	0.9039	23.0867
**PNLIP Inhibition**	0.0200	0.2258	0.6103	0.4934	0.1134	5.9101

[1]Model adequacy was verified using analysis of variance (ANOVA) by evaluating the model significance ($p<0.05$), lack-of-fit, and coefficients of determination (adjusted R^2, predicted R^2). Differences between adjusted and predicted R^2 below 0.2 were considered acceptable for good predictive performance. The Adequate Precision ratio was also examined to confirm a satisfactory signal-to-noise ratio (>4).

Although in a few cases the difference between adjusted and predicted R^2 slightly exceeded 0.2, those models were retained since all other statistical criteria were fulfilled (significant regression, non-significant lack-of-fit, and adequate precision >4).

TFC was not measured using analytical replicates and for that reason, its importance was set to 1 during optimization, ensuring that it did not dominate the desirability outcome.
